# Supplementary material for: Are carbon emissions trading and green financial instruments synergistic? -Comprehensive quantitative research based on content analysis
Source: PLoS One. 2024 Mar 7;19(3):e0298601. doi: 10.1371/journal.pone.0298601 (PMC10919601; doi:10.1371/journal.pone.0298601)
Supplement: S1 Data — (ZIP) [file pone.0298601.s001.zip › Data/地级市Z_F工作报告.zip!/╣π╢1⁄2/╔╪╣╪╩╨/└┤╫╘╣1⁄2╓┌║┼í╛┬φ┐╦╩2╛▌═°í┐_2018.docx]

各位代表：

现在，我代表市人民政府向大会作工作报告，请予审议，并请各位政协委员和其他列席人员提出意见。

2017年工作回顾

过去一年，在省委、省政府和市委的正确领导下，在市人大、市政协的监督支持下，全市上下以习近平新时代中国特色社会主义思想为指导，深入贯彻党的十八大、十九大精神，认真贯彻习近平总书记对广东工作的重要指示批示精神，落实新发展理念，坚持以供给侧结构性改革为主线，大力实施主动融入珠三角战略，突出“三大主题”工作和“三大重点”民生，加强谋划，攻坚克难，狠抓落实，经济社会持续健康发展。2017年，全市地区生产总值1338亿元（预计数，下同）、增长6%，固定资产投资736亿元、增长5%，地方一般公共预算收入88.7亿元、增长6.2%。

——发展质量取得新提升。规上工业综合经济效益指数261%，利润总额增长56%。生产性服务业增加值占GDP比重达43%。来源于韶关的财政总收入、税收收入分别增长17.2%和10.2%，分别高出上年17.5个和7个百分点。非税收入占比39%，比上年降低1.5个百分点。

——城市提升取得新成绩。实施城市提升项目63个，完成投资56亿元。芙蓉新城实施项目33个，完成投资62亿元、增长24%。莲花大道一期通车，芙蓉隧道贯通，江湾大桥动工建设，“三江六岸”实现亮化提升，城市绿化、美化、净化和畅通水平不断提高。

——深化改革取得新亮点。供给侧结构性改革扎实推进，淘汰落后钢铁产能112万吨，居全省前列。商事制度“马上办”模式走在全省前列。完成市级行政服务大厅升级改造，建成“一门式一网式”政务服务平台，进驻服务事项979项、占比达95.5%，实现“一站受理、一窗通办”。在全省率先启动应收账款融资服务。

——新农村建设取得新成果。2424个自然村完成阶段性农村人居环境综合整治。278个省定贫困村创建新农村示范村工作实现“四个100%”。1.06万户3.36万人实现脱贫，获得全省脱贫攻坚综合评价地级市第一名，我市“六种帮扶模式”经验被国务院扶贫办推广。翁源县南塘村被评为中国美丽休闲村。

——生态文明建设取得新成效。全市森林覆盖率75.05%、有林地面积1911万亩、活立木蓄积量9054万立方米，继续稳居全省首位。在全省开创林业碳普惠制碳汇上市交易先河。饮用水源水质达标率100%，空气质量继续保持优良。荣获“中国十大品质休闲城市”称号。

——社会民生取得新进步。全市民生投入238.8亿元、增长19.1%，占财政总支出76.8%。城乡居民人均可支配收入达2.18万元，增长9%。企业职工基本养老保险实现省级统筹，城乡居民基础养老金最低标准和低保人均标准进一步提高。完成棚户区改造3202套。全面启动农村危房改造5600户，竣工3171户。

一年来，市政府主要抓了以下九方面工作：

一、强力推进产业攻坚，转型升级行稳致远

大力推动钢铁、有色冶金等传统优势产业转型升级。在大力淘汰落后钢铁产能的同时，新增特钢产量20万吨，特钢产值增长29.3%；钢铁和有色金属两大传统产业工业增加值占比由14.5%调整到16.7%。设立企业技改项目风险补偿基金，宝武（韶关）特钢、大宝山铜硫选厂等一批工业技改项目建成投产，全年完成技改投资60亿元。大力培育发展先进装备制造、旅游文化、大数据、商贸物流、医药健康等新兴支柱产业。装备制造业增加值增速高于全市规上工业增加值2.7个百分点，我市机械基础零部件集群纳入国家第三批创新型产业集群试点。中车株洲智轨生产基地、亿航智能无人机等一批重大项目签约。“华南数谷”大数据产业园成功授牌，引进华为参与智慧城市建设。大力发展民营经济，全市私营企业数量增长12.8%。　　现代服务业增加值增长8.5%，服务业占GDP比重达49.7%。全面提升企业科技创新能力，高技术制造业增加值20.5亿元、增长15.2%。新增高新技术企业36家，主营业务收入5亿元以上工业企业研发机构实现全覆盖，规上工业企业设立研发机构比例提高到20%。

二、狠抓园区提质增效，工业经济固本培元

推动韶关高新区创建国家高新区，支持南雄创建省级高新区，着力推进莞韶园和华南装备园开发建设。出台产业园区加快发展及招商引资政策，改革产业园区（集聚地）工作绩效考核制度，赋予华南装备园管委会市级管理权限。全市产业园区新开工项目174个、建成投产项目59个，完成固定资产投资125亿元，园区工业投资占全市比重达62.2%，实现税收增长11.6%。加强产业服务配套建设，新增省市级工程技术研发中心63家，新增省级孵化器、省级众创空间各2家，增量位居全省前列。实现国家级科技企业孵化器和国家产品质量监督检验中心零的突破。加强招商引资工作，全市引进产业项目115个，总投资326.6亿元，其中亿元以上项目51个。

三、深化重点领域改革，发展环境不断优化

始终把深化改革作为破解发展瓶颈的关键，突出抓好供给侧结构性改革，拆除6家企业落后炼钢设备，实现8户国有特困企业脱困。净化解商品房65.8万平方米，库存面积下降37.7%。深化“放管服”改革，取消、下放市级行政许可事项105项，清理规范中介服务事项30项。行政审批标准化建设加快，投资项目审批效率不断提高。完成市县镇村四级服务平台建设。实施工商登记“多证合一、一照一码”，新登记市场主体2.44万户。完善市领导班子成员挂点联系企业制度，出台降低制造业企业成本32条，取消或停征41项行政事业性收费，积极推进直供电试点，统筹降低企业社保缴费比例，全年减轻企业负担43亿元。成功争取乳源县少数民族地区企业所得税优惠政策延期至2025年。

四、持续推进城市提升，城市面貌不断改善

加强规划引领，我市城市总体规划成为全省首个批复至2035年的城市总体规划，建成市区“多规合一”基础数据展示和管理平台。完成韶关学院韶州师范分院迁建主体工程。实施市区道路“白改黑”工程35.5公里，完成解放路断面改造和韶关大道改造一期工程，整治6个主要交通节点，新建2座人行天桥，建成小岛9.9公里慢行系统。加固市区河堤9.3公里，建成东河片区截污管网10.9公里。改造提升21个农贸市场，完成41个“五小”民生工程，城市功能不断完善。开展芙蓉新区建设“百日攻坚行动”，新白线、市妇幼保健计生服务中心等项目破土动工，完成百旺路、滨江路和综合客运枢纽主体工程建设。新区征地拆迁取得突破性进展，拆迁房屋17万平方米，交地5000亩。入选全国老旧小区改造试点城市。投入5.4亿元创建全国文明城市，顺利通过创文国检测评，“善美之城”品牌深入人心。

五、谋划推进全域旅游，旅游经济持续旺盛

大力实施国家全域旅游示范市创建三年行动计划，启动全域旅游发展规划编制和全域旅游资源普查工作，南雄被列入第二批广东省全域旅游示范区。突出抓好大丹霞、大南华、大南岭、大珠玑四大重点景区建设，全市旅游项目完成投资13.6亿元，建成旅游集散中心5个、旅游公路50公里和旅游厕所207座，荣获“全国厕所革命先进市”称号。推动旅游融合发展，大力发展乡村旅游，开展南粤古驿道定向大赛韶关站等系列户外活动，抓好红军长征粤北纪念馆和南雄原苏区县红色旅游开发建设。提升旅游服务水平，实施红黑榜制度，启动智慧旅游公共服务信息平台建设。新增2家国家AAA级旅游景区。全市接待游客4237万人次、增长18%，旅游收入389.9亿元、增长20%，带动营利性服务业增加值增长14%。

六、大力推进县域发展，城乡区域更加协调

始终把加快县域发展作为我市实现振兴发展的关键。积极推进国家新型城镇化综合试点工作。大力改善县域基础条件，武深高速仁化段建成通车，汕昆高速韶关段进展顺利，韶新高速动工建设。新增和改造电网线路1318公里。实现村村通光纤，建成南雄原中央苏区农村超高速无线局域网。完成中小河流治理605公里。超额完成省下达的水田垦造任务，高标准基本农田建设走在全省前列。加快推动县域经济发展，支持打造特色优势产业，实现县域生产总值748亿元、增长5.9%，其中农业增加值145亿元、增长4.5%。省级现代农业“五位一体”示范基地实现县域全覆盖。农村土地承包经营权确权登记颁证完成96.4%，排名全省前列。开展13个市级示范特色小镇建设，江尾兰花小镇被列入省特色小镇创建工作示范点。新农村建设扎实推进，实施6995个自然村人居环境综合整治工作。

七、大力加强生态建设，环境质量持续改善

落实主体功能区规划，开展生态红线划定工作，启动粤北生态特别保护区规划。广东绿色生态第一市建设扎实推进，完成造林40万亩、森林碳汇造林6.2万亩，完善提升生态景观林带38公里，新增森林公园11个、湿地公园1个。完成国有林场改革。始兴深渡水瑶族乡、乐昌九峰镇、南雄帽子峰镇入选首批“广东省森林小镇”。在全省率先建成森林航空消防基地。推进大气污染防治，加强施工扬尘、工业和机动车排污治理，整治“小散乱污”企业51家、关闭25家，市区空气质量优良率89.8%。加强水污染防治，全面落实河长制，完成48个畜禽养殖污染减排项目，水环境质量保持良好。推进土壤污染综合防治先行区建设。建成能耗大数据监控平台，完成年度减排目标任务，通过国家节能减排财政政策综合示范城市三年总体考核。

八、矢志不渝改善民生，社会事业全面发展

市政府承诺为民办理的10件计20项实事，大部分已完成和基本完成。全市有劳动能力贫困户人均可支配收入达9272元，贫困户100%纳入大病救助范围。稳步推进教育现代化先进市建设和教育综合改革，扎实推进省市共建韶关学院，武江区被授予“广东省推进教育现代化先进区”。深入推进卫生强市和医药卫生体制综合改革，全市23家公立医院全面取消药品加成，市县镇三级医院全面参与医联体建设，完成13家乡镇卫生院标准化和353间卫生站规范化建设。全面实施机关事业单位养老保险制度改革。实现跨省异地就医直接结算，基本医疗保险参保率达98.1%。成立市流浪乞讨人员安置中心并设置应急救助基金。深入实施“双百人才工程”，引进94名紧缺适用人才，选拔出首期享受市政府特殊津贴人才111名。城镇登记失业率低于3.5%。建成745个行政村（社区）基层综合性文化服务中心，完成广富新街、斌庐等历史文化街区遗址修缮工作，举办南国书香节、市第五届戏曲曲艺花会等群众文化活动，大型现代采茶戏《枫树湾近事》在第十三届广东省艺术节展演。我市运动员在十三届全国运动会中荣获7枚金牌，在省第四届体育大会上总分位列粤东西北第一。获全省粮食安全责任制考核优秀等级。“平安韶关”创建成效显著，实现“四个不发生”目标，十九大安保工作获省表彰，群众饮食用药安全得到保障，安全生产形势总体稳定。外事侨务、民族宗教、国防教育、人民防空、应急管理、防灾减灾、打击走私、档案管理、妇女儿童、残疾人、地方志、红十字会等事业取得新发展。

九、不断厚植勤廉之风，政府建设有效加强

扎实推进“两学一做”学习教育常态化制度化。坚决落实市委重大决策部署，坚持向市人大及其常委会报告工作、向市政协通报工作情况，注重发挥民主党派、工商联、无党派人士和人民团体的作用。办理人大议案1件、代表建议81件、政协建议案5件、政协提案214件，办复率100%。积极推进法治政府建设，制定政府起草地方性法规草案和政府规章程序规定，选聘新一届市政府兼职法律顾问。积极推进行政机关负责人出庭应诉。开展芙蓉新区正向激励和反向问责试点。加强监察工作，稳步推进审计制度改革和审计全覆盖。依法依规做好统计工作。积极防范化解政府债务风险。全面实施“七五”普法工作，广大市民的法治意识不断提升。认真落实中央八项规定精神，加强廉洁政府建设，严肃查处了一批损害群众利益的突出问题。

各位代表！这些成绩的取得，最根本的是有习近平新时代中国特色社会主义思想的指导，有省委、省政府的坚强领导，有市委的正确领导，也离不开市人大、市政协的监督支持，离不开历届党委政府打下的坚实基础和全市干部群众的艰苦奋斗，离不开社会各界的关心支持。在此，我谨代表市人民政府，向全市人民、各位人大代表、政协委员，向各民主党派、各人民团体，向驻韶中省单位、人民解放军、武警部队官兵，致以崇高敬意！向历届老领导老同志、所有关心支持韶关经济社会发展的各界人士，表示衷心感谢！

在看到成绩的同时，我们必须正视我市发展不平衡不充分的问题，主要是：经济总量不大，产业结构不优，实体经济薄弱，工业发展短板突出；“两个单一”的历史困局没有有效破解，新的发展动力没有形成；县域经济薄弱，土地、资金、人才和环境容量等制约因素仍然存在；城乡规划建设管理滞后，芙蓉新城建设亟待提速，街区核心历史文化价值和特色需要进一步挖掘、保护和展现；距离全面建成小康社会指标体系差距比较明显，2017年地区生产总值没有实现预期目标，落后预期目标2个百分点；基本公共服务均等化水平较低，在就业、就学、就医、养老等方面与群众对美好生活的向往仍有较大差距，等等。对此，我们将勇于直面问题，进一步增强责任感、使命感和紧迫感，切实解决好制约我市发展的突出问题。

2018年工作安排

2018年是贯彻落实党的十九大精神的开局之年，是改革开放40周年，是决胜全面建成小康社会、实施“十三五”规划承上启下的关键一年。今年政府工作的指导思想是：全面贯彻党的十九大和中央经济工作会议精神，以习近平新时代中国特色社会主义思想为指导，按照省委、省政府和市委的统一部署，坚持稳中求进工作总基调，牢固树立新发展理念，紧扣社会主要矛盾变化，贯彻构建现代化经济体系和高质量发展的要求，坚持以供给侧结构性改革为主线，坚决打赢防范化解重大风险、精准脱贫、污染防治“三大攻坚战”，坚持主动融入珠三角战略，突出重大基础设施、产业园区、中心城区、全域旅游、乡村建设、生态建设六大发展重点和重大社会民生工作，统筹稳增长、促改革、调结构、惠民生、防风险各项工作，着力补短板、强弱项、解难题、重落实，全力加快振兴发展。

今年全市经济社会发展的主要预期目标是：地区生产总值增长7.5%，人均生产总值增长6.5%，工业增加值增长8.5%，固定资产投资增长12%，社会消费品零售总额增长8%，一般公共预算收入增长6%，城乡居民人均可支配收入增长与经济发展同步，主要污染物总量减排、居民消费价格指数和计划生育等约束性指标完成省下达目标任务，城镇登记失业率控制在3.5%以内。

围绕以上目标，我们将重点做好以下九个方面工作：

一、突出发展实体经济，推动经济量质齐升

坚持把发展实体经济作为经济建设的首要任务，突出工业提质增效，强化科技创新，大力培育发展新产业新业态新动能，推动实体经济做大做强。

强力推进产业集群发展。强化企业挂点帮扶，选取50家存量优势企业进行重点培育，助推企业规模和效益双提升。制定工业增长点清单，确保新增规上企业30户。实施企业倍增计划，首批选取100家优质制造企业，通过政策扶持、政府服务，引导企业加强技改、增资扩产，推进产能倍增。依托优势产业和龙头企业，重点推进先进装备制造、生物制药、大数据等产业集群发展。大力发展特钢产业，组建特殊钢及汽车零部件产业发展联盟，打造高端优特钢生产基地。充分发挥特钢等材料优势，力争引进亿元以上装备制造项目15个，推动液压件、汽车零部件、发配电设备、矿山工程机械等转型升级，发展成套装备及整机设备，抱团做大做强装备产业集群。助推乳源东阳光公司建设成我市县域经济首个百亿级特色产业集群。精心培育一批代表科技前沿的产业项目。开展招商引资年活动，整合招商引资力量，实施精准招商，全市新签约产业项目100个以上、总投资300亿元以上，其中落地项目70个以上、总投资200亿元以上。

全力打造产业发展平台。深入实施园区三年提升计划，完成基础设施投资12亿元，实现园区工业投资增长20%，规上工业增加值增长12%，占全市工业增加值比重提升至40%以上。推进莞韶园开发建设，实施甘棠片区提升计划，开工建设莞韶城三期，推动莞韶园与周边镇村融合发展。加快华南装备园开发，尽快打通到市区的主通道，建设6条主干道和综合服务大楼，统筹推进韶钢与周边村庄的区域规划建设。深化与广州、深圳、东莞的产业合作，推进珠江东岸产业合作区建设。以对接广汽集团为契机，力争打造汽车零部件生产基地。加大引进深圳科技产业力度。依托莞韶产业共建，全力推动县域园区加快发展，完善园区生活配套和公共服务平台。发挥市区教育组团的优势，加快谋划市区新的高科技产业平台，打造新的增长极。加强园区要素保障，着力化解“项目等地”的困局，补齐园区环境、设施、功能等短板。

大力推进民营经济发展。扩大民间资本投资范围，积极引导民营企业参与PPP模式开发建设运营。贯彻落实好国家新修订的中小企业促进法，落实好我市发展民营经济49条、降低制造业企业成本32条。完善中小企业服务体系，进一步做强市县两级“1+10”示范性公共服务平台。拓宽企业融资渠道，完善“助保贷”政策，引导金融机构扩大信贷投放，推广应收账款融资业务，着力解决银行转账限制和贷款到期“先还旧再借新”的问题。推进创业投资基金建设。充分发挥商会、协会的作用，做好“安商”“稳商”工作，畅顺政府与企业沟通渠道。抓好“个转企、小升规、规改股、股上市”工作，新增“四上企业”150家，民营经济增长4%。

深入实施创新驱动战略。加快创建国家级高新区，支持县域园区创建省级高新区，力争新增高新技术企业50家以上。引导各类主体建设科技企业孵化器，加快莞韶双创中心、创智城、众投邦加速器的建设运营。推动企业建立研发机构，巩固主营业务收入5亿元以上工业企业建立研发机构全覆盖成果，1亿元以上工业企业建立研发机构达到30%，规模以上工业企业建立研发机构达到25%，全年新增企业研发机构40家以上。深化政产学研合作，重点加快高品质金属材料研究院、华实现代农业创新研究院等新型研发机构建设。争取中科院韶关实验室申报国家级检测平台，创建国家级产业计量平台。

二、大力发展现代服务业，打造新的经济增长点

增强服务业对经济发展的支撑作用，重点推动全域旅游、商贸物流、现代金融等行业提档加速。

大力发展全域旅游。突出“四大景区”龙头带动效应。引进战略投资者共同开发建设丹霞山，“打通南门，联通北门”，加快环丹南部片区开发，丰富旅游产品种类，提升核心产品竞争力，全面改进服务质量。围绕“一寺两院”建设，加强周边环境整治，展示禅宗文化特色，推动大南华整体提升。结合粤北生态特别保护区和环南岭生态旅游公路规划建设，加强西京古道保护利用，统筹大南岭景区建设。做好梅关古驿道提升工程，充分展示姓氏迁徙文化内涵，加快大珠玑景区建设。高质量建设红军长征粤北纪念馆，加快城口红色小镇建设，做好红色文化遗产的保护和利用，打造红色文化教育基地。围绕四大景区和周边重要景点之间的互联互通，谋划建设一批全国一流、广东领先的旅游公路，开工建设旅游公路100公里。加快“九龄故里·百里画廊”示范线路等13个旅游重点项目建设，完善旅游服务中心、标示导向、停车场、旅游厕所等配套设施。加强旅游资源整合和业态布局，促进旅游与文化、农业、体育等产业融合发展，打造10条乡村旅游精品线路，新增省级休闲农业和乡村旅游示范镇2个、示范点4个。整合提升全市旅游节庆活动，统筹策划“活力之旅、善美韶关”系列活动，开展10场旅游招商推介活动，打造韶关特色旅游文化品牌。实现接待游客、旅游收入分别增长10%以上和12%以上。

大力发展商贸物流。加快重大物流平台建设，推动粤北国际物流园建设取得实质性进展。规划建设韶钢至乌石的铁路货运通道，推进白土港口建设，积极发挥“铁海联运”作用。鼓励电子商务企业集聚发展，推动市电子商务产业园建设。积极发展农村电子商务，完善县镇村电商服务体系。开展“快递下乡”工程，提高乡镇快递网点覆盖率。充分利用乳源县税收优惠政策，规划建设总部经济园区。促进批发业加快发展，实现批发零售业增加值增长6.5%。培育新增限上商贸企业20家以上。提升韶南大道汽车商贸产业带。培育外贸新增长点，拓展韶关跨境电商清关中心业务，推进进出境快件监管场所项目建设。

大力发展现代金融。加大金融、科技、产业融合力度。支持金融机构为科技创新创业企业提供专业化的高效信贷服务，实现金融业增加值增长8%以上。整合组建市级基础设施和产业基金，带动社会资本进入基础设施和产业投资领域。支持引导我市现有产业基金加快投放。大力开展金融招商，积极引进各类金融机构进驻。全面完成农信社改制、组建农商行工作。强化政银企合作，出台政策性担保机构担保费补贴政策，探索开展“政银担”业务。加快天使母基金项目落地，推动知名创投企业进驻。

三、坚持以项目为抓手，实现投资有效突破

开展项目建设攻坚年活动，聚焦项目落地建设和投产达效，全年计划安排重点建设项目144个，完成投资381亿元。

狠抓产业项目建设。千方百计扩大我市产业项目有效投资，加快76个产业重点项目建设、完成投资133.4亿元。推动韶钢技改、韶铸搬迁改造等工业技改项目建设，加快去年集中动工建设的72个产业项目尽快投产达效。着力抓好中色南方稀土、白云电气、忠信集团等项目开工建设。推进云门山旅游度假区、黄金（商创）小镇、华南农产品交易中心二期等服务业项目建设。

加快交通基础设施建设。加快构建“八高四铁二航”的交通综合体系，确保武深、汕昆高速公路建成通车；加快韶关丹霞机场、韶新高速、北江航道扩能升级工程建设。加快推进韶柳铁路、赣韶铁路复线、韶连高速、雄信高速、云轨项目、新丰至从化快速交通和G323线公路改造等项目前期工作。开展公路安防整治234公里。开工建设机场进场道路和韶关南高速出口至马坝人遗址改扩建工程。

推进水利能源等项目建设。大力推动华电南雄“上大压小”热电联产工程、上庙水库等13宗重点项目建设。推进小型农田水利重点县和仁化水系连通试点县建设，完成30宗病险山塘加固工程和204.8公里山区中小河流治理项目。推进500千伏丹霞变电站工程前期工作，实施好1148宗新一轮农村电网改造升级工程。加快推进市区第三污水处理厂、镇村污水处理设施及管网项目建设，实现乡镇生活污水处理设施全覆盖。

加强民生项目建设。建立稳定财政投入机制，支持教育、医疗卫生、文化体育、保障性住房等社会事业。突出21个民生类重点项目建设，完成投资58.4亿元。继续推进市区教育资源整合，完成市二中、曲仁中学合并迁建及浈江区风采实验学校等项目建设。加快市妇幼保健计生服务中心、市第一人民医院迁建、粤北二院综合大楼及南雄、乐昌、翁源第二人民医院等医疗卫生类重点项目建设。推动韶州文化广场“一中心三馆一剧院”开工建设。推进南水供水工程、公安消防新指挥中心等民生工程建设。

四、全面深化改革，争创发展环境新优势

坚决破除一切不合时宜的体制机制，打造服务意识更强、行政效率更高和营商成本更低的营商环境，增强发展环境吸引力竞争力，激发全社会创造力和发展活力。

大力推进基础性关键性领域改革。继续深化供给侧结构性改革，重点在“破、立、降”上下功夫，实现全市国有“僵尸企业”出清。出台深化国资国企改革方案，推动国企按竞争性和准公共性分类管理，引导和鼓励非国有资本以多种方式参与国企改革，加大国资国企整合重组力度。深化市与区的管理体制改革，赋予区更大的自主权，推动区和市区范围的镇（街）成为更重要的发展主体。改革完善园区管理体制，科学赋予管委会开发建设所需的管理权限。积极稳妥推进财税、金融、社保、教育、医疗等重点领域和关键环节的改革。

深化放管服改革。升级完善市级“一门式一网式”政务服务平台，加快推广至县（市、区）和镇村。继续精简行政审批事项，再造审批流程，积极应用信息化手段，推进行政许可标准化和协办联办，确保不涉及新增建设用地投资项目审批控制在28天内。深化中介服务市场化改革，加快中介超市建设。深化商事制度改革，全面推广电子营业执照和全程电子化登记管理，大力推行“马上办”“跑一次”“零见面”服务模式。继续推进“减证便民”行动。推进“双随机、一公开”监管全覆盖，探索实施跨部门“双随机”联合检查。

降低企业经营成本。聚焦企业社保费用、涉企收费、物流、融资、用地、用能和制度性交易成本，研究推出新的降成本措施，为企业减负30亿元以上。继续推进大用户直购电和售电侧改革。实施涉企收费目录清单管理。建立完善高成长小微企业名录库，落实中小微企业贷款贴息制度。充分利用多层次资本市场，支持企业通过发行债券、资产证券化等方式直接融资，降低企业融资成本。加强公共就业服务，推动校企订单式服务和联合培养，解决企业劳动力紧缺问题。采取以租代售等多种灵活供地方式，降低企业用地成本。

大力推进人才强市。加大资金投入，确保人才资源开发专项资金1亿元以上。继续实施“双百人才工程”，发挥首期享受市政府特殊津贴人才的示范带动作用，加大引才力度。实施人才安居工程，投资2亿元建设人才公寓，规划建设国际高端人才社区。开辟人才绿色通道，扶持产业科技人才及创新创业人才团队1000人，采取有力措施引导在韶就读大学生留韶工作，鼓励大学生在韶创业就业。扩大技能人才有效供给，落实职业技能晋升补贴政策，实施基层医疗卫生“千人计划”。发挥驻外机构、商会协会的桥梁作用，促进招才引智。

五、全力加快扩容提质，提升城市功能和品质

坚持人民满意的工作导向，继续加大城市提升工作力度。以片区为单元狠抓城市连片提升，提升中心城区竞争力、资源集聚力和辐射带动力。

优化城市空间布局。深化城市规划，优化中心城区布局，实现中心城区控规“一张图”。今年重点围绕五大片区，打造城市提升的示范和标杆。高标准建设芙蓉新区，完成投资70亿元，打造“产城融合的现代化新城区”。小岛片区按照“风情小岛、韶关记忆”主题，打造“特色文旅商业区”。以韶州公园为“绿心”，将韶州公园片区打造为“宜居宜业的现代城市生态生活区”。依托现有大型商住项目集聚效应，将芙蓉山北部片区打造为“新型商贸集聚区”。支持曲江新城建设，推动曲江片区建设“工贸旅集约发展区”。同时，提升城郊周边镇和城边村的规划建设水平，打造优美、特色、活力城郊。　　构建畅顺城市交通体系。树立路畅城靓理念，重点实施10个交通节点和10条重要干线周边景观环境整治，加快市区高速出口的整体风貌改造。在环城高速的基础上，贯通浈、武两区的内环，建设生态路穿越莲花山隧道连接莲花大道的中环，加快构建城市道路三环。加快推进曲江大道、江湾大桥、芙蓉大道、新白线等交通干线建设，建设城区智能轨道交通。通过“建、改、拓、延”相结合，加强交通骨干网络和支次路网衔接，改造7个交通堵点，打通6条断头路。

构建优美城市生态景观。充分发挥资源禀赋优势，打造现代山水名城。加快城区与景区联动发展，规划建设舜帝森林公园，谋划丹霞山西南门连接市区、南华寺至丹霞山南门的旅游通道，实现融山于城、融景于城。推进“三江口”建设，打造城市客厅。加强城市绿道建设。启动“小山小湖”规划建设，续建小公园、小绿地30个和亲水平台3个，打造“推窗见绿、开门见园”的生态休闲公园体系。大力推进滨江景观带、芙蓉中央公园、盆景山公园、林桥公园建设，活化新城水系，绿化破损山体，促进城市与生态保护融合发展。

强化城市经营理念。合理布局城市商业用地，促进土地保值增值。加强土地储备，开展划片整体收储试点工作，整治一批国有低产低效土地，集中盘活政府性资金资产资源。树立城市更新理念，用好国家老旧小区改造试点城市政策，开展5个老旧小区改造试点，鼓励企业腾笼换鸟、优二进三，将老旧厂房改造为文化创意、电子商务或企业办公用房。推动都市型产业优先在芙蓉新城、三江六岸优质地段布局和发展，统筹做好高铁站前片区、“华南数谷”核心区等重点片区规划建设及招商工作，提升城市发展内生动力。制定促进楼宇经济发展政策，引导各类商务楼宇形成产业特色，支持企业来韶建设总部大楼。

提升城市治理水平。加大文明城市创建力度，擦亮“善美之城”品牌。加强对裸露地管理，深入推进拆违整治工作。狠抓市容市貌整治，突出整治城市“六乱”，重点在城市立面、公共空间、渣土扬尘、临街门店、建筑材料堆场、废品回收场地等方面发力，加强农贸市场周边环境整治。建设智慧城市调度指挥中心。加强市区停车场规划建设，综合整治交通秩序。推行社区“网格化”服务管理，严格落实“路长制”“楼长制”“门前三包”责任制，提高精细化管理水平。

六、着力实施乡村振兴战略，统筹城乡一体化发展

实施乡村振兴战略是新时代“三农”工作总抓手，按照产业兴旺、生态宜居、乡风文明、治理有效、生活富裕的总要求，坚持农业农村优先发展，加快实现农业强、农村美、农民富。

构建现代农业产业体系。深化农业供给侧结构性改革，推进农业由增产向提质转变。制定实施现代农业产业发展规划，着力构建农村一二三产业融合发展体系。推行农村土地三权分置，推进农村集体产权制度改革，全面完成农村土地确权颁证。推动资本、人才下乡和企业入村，引导社会资本进入农业领域，促进农业生产方式由“单家独户”向“园区集群”转变。重点打造仁化国家现代农业示范园区、粤台农业合作试验区翁源核心区，建设一批万亩农业产业基地。积极培育新型农业经营主体，新增市级示范家庭农场50家以上、省市级农民合作示范社20家以上、市级农业龙头企业15家以上。加大生态原产地产品保护示范区和出口食品农产品质量安全示范区建设投入，力争建成国家级、省级示范区4个。大力培育田园综合体，每个县创建一个集“山水田园湖林路+特色农业+N”的现代农业博览园。加快推进13个特色小镇建设。发展农产品深加工和农村电商，多渠道增加农民收入。加强品牌保护和产品溯源工作，培育省级农业名牌产品10个以上、“三品”认证产品20个以上。办好第28届中国兰花博览会，举办全市首届生态农业博览会。

加快社会主义新农村建设。以集中连片推进的方式全面开展新农村建设，建设美丽宜居乡村。重点抓好环丹霞山片区、市区城郊农村片区、南雄古驿道新农村连片示范工程，推进278个省定贫困村建设新农村。着力把全市重要交通干线沿线和主要景区、中心镇周边的1089个自然村打造成为新农村样板村。加强农村人居环境综合整治，深入推进城乡清洁工程，完善农村生活污水处理和消防设施，实现农村生活垃圾收运处理体系全覆盖。改善乡村基础条件，加强“四好农村路”建设，完成300公里自然村公路路面硬化，新增和改造10千伏及以上供电线路1152公里。加强乡村文明建设，推动社会治理和服务重心向基层下移，推动自然村成立村民理事会，鼓励乡贤反哺支持家乡建设，构建自治、法治、德治相结合的乡村治理体系。

坚决打好精准脱贫攻坚战。坚持把提高脱贫质量放在首位，注重扶贫同扶志、扶智相结合，激发贫困人口内生动力。着力构建党政、行业、社会“三位一体”大扶贫工作机制，压实县镇村主体责任，巩固预脱贫的2.73万户6.45万人，确保再脱贫0.76万户2.54万人。突出产业扶贫，加快6个100万亩山地特色产业基地建设，发展“企业+基地+合作社+贫困户”等多种帮扶模式，推动光伏、旅游、电商等项目落实到村到户。落实危房改造、就业、教育、医疗、低保兜底等政策。

七、坚持绿色发展，开启生态文明建设新航程

良好的生态环境是韶关最大的发展优势之一。践行绿水青山就是金山银山的理念，牢牢把握生态发展定位，努力在高水平保护中实现高质量发展，争当全省绿色发展排头兵。

坚决打好污染防治攻坚战。实行最严格的生态环境保护制度，着力解决突出环境问题。严格落实河长制，开展北江、浈江、武江、新丰江等跨区域河流水环境联防联治，加强农业面源污染防治，抓好规模化畜禽养殖场治理，确保饮用水水源水质安全。加强工业废气、露天焚烧、施工扬尘、机动车尾气的治理，确保城市空气质量优良率达90%以上。抓好全国土壤污染综合防治先行区建设，全面排查工业固体废物堆存场所，推进大宝山、凡口等周边土壤治理修复，实行建设用地土壤环境质量强制调查评估。完善污染预警机制，加大对违法排污、挖山、采砂采矿的查处整治力度。

加大生态环境保护和建设力度。深入落实主体功能区规划，完成全市生态保护红线划定工作，严格执行林地定额制度。规划建设粤北生态特别保护区，着力构建广东北部山区生态功能区新格局。主动谋划建设国家公园。积极创建国家森林城市，丰富植物品种，加强林相改造，由过去较为单一的绿化向美化彩化转变。扎实推进广东绿色生态第一市建设，开展森林抚育60万亩、碳汇造林5万亩，新建提升生态景观带93公里，新增森林公园2个、湿地公园1个。

推进绿色低碳发展。探索设立市绿色发展基金。加快推进循环经济环保园一期项目建设。加强绿色技术创新体系建设，加快节能新技术、新材料、新设备的推广应用，促进钢铁、冶金、电力、建材等传统产业节能改造。强化重点用能单位节能管理，建立行业领域节能“领跑者”标准制度，整体提升全社会能源利用效率。倡导简约适度、绿色低碳的生活方式，推进节约型机关、绿色家庭、绿色校园、绿色社区建设，增强全民环保意识。

八、着力保障改善民生，提升群众获得感

满足人民群众对美好生活的需求，努力为群众提供更好的教育、更稳定的工作、更满意的收入、更可靠的社会保障、更高水平的医疗服务、更舒适的居住条件、更丰富的精神文化生活。

推进教育优先发展。深化教育领域综合改革，争创省推进教育现代化先进市，实现省推进教育现代化先进县（市、区）全覆盖。积极稳妥推进中小学教师“县管校聘”管理改革。深化省市共建韶关学院，推进大学科技园建设，支持高校内涵发展。重视学前教育安全管理和优化布局，加强未成年人思想道德建设，创建70所文明校园。加快推进韶关智慧教育项目建设，培育发展智慧教育产业。深化产教融合、校企合作，争创省现代职业教育综合改革示范市。

推进健康韶关建设。深化医药卫生体制改革，加强省卫生强市建设，做好人口计生工作，推进医联体、医疗集团建设，扩大县域医疗服务共同体试点。提高基层医疗卫生服务水平，用5年时间引进和培养1500名以上基层医疗卫生实用型人才。推行分级诊疗制度，继续扩大家庭医生签约服务覆盖面。推进“医养结合”的健康养老服务，试点特困人员供养服务机构公建民营社会化改革，确保每千名老人拥有35张养老机构床位。

推进文体事业发展。进一步加大资金投入，加强小岛片区文物古迹修缮保护和活化利用，实现基层综合性文化服务中心全覆盖；系统挖掘梳理红色文化、工矿文化、丹霞文化、禅宗文化、珠玑文化、客家文化、瑶族文化等历史文化资源，推出一批研究和活化利用成果，擦亮文化品牌；加大政府购买服务力度，推出一批群众喜爱的文化产品，举办好南国书香节、纪念改革开放40周年系列活动等文化活动。擦亮广东户外运动天堂品牌，加强公共体育场地设施建设，做好市体育中心项目前期工作，打造城市社区15分钟健身圈。

提高社会保障水平。实施积极的就业政策，大力推动“双创”，支持创业就业孵化基地建设，促进异地创业和务工人员返乡创业就业，力争城镇新增就业3.2万人，城镇失业人员再就业2.8万人，促进创业1600人。加强社会保险扩面征缴，兜牢社保民生底线，全面实施全民参保计划，提高医保待遇水平，开展二次医疗救助。加快建立多主体供应、多渠道保障、租购并举的住房制度。

推动社会治理创新。推进“数字政府”建设，应用大数据提升社会治理智能化水平。构建社会信用体系和市场监管体系。发挥社会组织积极作用。依法及时就地解决群众合理诉求。筑牢数字化立体化治安防控体系，开展打黑除恶专项行动，加强“扫黄打非”工作，严厉打击黄赌毒和盗抢骗等违法犯罪，全面推进全民禁毒工程，建设“平安韶关”。加强安全生产管理，推进消防安全“网格化”建设，加强地质灾害防治，加快实施南水水库泄洪渠弃渣整治工程，防范重特大事故发生。强化食品药品安全，打造一批示范性“放心餐馆”“放心药店”。落实粮食安全政府责任制。

今年市政府重点办好十件民生实事：1.推进医疗基础设施建设；2.加强城乡生活垃圾处理；3.继续推进南水供水工程建设；4.加强保障性住房建设；5.推进“厕所革命”；6.开展“明厨亮灶”工程；7.推进市区风度书房建设；8.提供公安便民服务；9.实施市区城乡结合部路灯安装升级工程；10.建设气象科普场所。

九、坚持实干导向，打造为民务实清廉的法治政府

打铁必须自身硬。坚持以实干创业、结果说话为工作导向，大兴实干之风，恪守发展之责，提升政府依法履职能力和水平，建设人民满意的政府。

依法行政，规范履职行为。自觉接受党委领导，围绕党委重大决策和部署抓好落实。自觉接受人大及其常委会和人民政协、新闻舆论、人民群众的监督，认真办理人大代表议案、建议和政协委员提案，加强12345政府服务热线建设，开展“我向市长说句话”网民建言征集活动，积极听取社会各界的意见建议。健全政府法律顾问工作机制，实施重大行政决策目录管理。健全部门权责清单动态管理机制，规范权力运行。坚持严格规范公正文明执法。加强行政复议和行政应诉，完善行政争议协调化解机制。

实干兴政，提高执行能力。施政之要，贵在落实，重在实干。坚决纠正调门高、只表态、不行动、不作为、乱作为、慢作为的不良作风，以更强担当精神履职尽责。实施市政府系统争先进位抓落实评价制度，努力形成“比学赶超、争先进位”的良好工作局面。建立科学高效的督查督办制度，强化工作追责问责。健全鼓励创新、容错免责机制，锻造一支想干事、敢干事、干成事的干部队伍。

从严治政，加强廉政建设。深入学习贯彻落实党的十九大精神，牢记“五个必须”，强化“四个意识”和“四个自信”，开展“不忘初心，牢记使命”主题教育。认真落实党风廉政“两个责任”，坚持不懈贯彻执行中央八项规定精神。严格执行监督制度，将“三重一大”事项纳入风险和可控性评估管理，推进廉政风险防控工作全覆盖。继续整治“四风”突出问题，坚决杜绝“门好进、脸好看、话好听但事难办”的现象。充分发挥审计监督和保障作用，加强审计整改工作，坚决纠正年年审、年年一个样的问题。深化标本兼治，加大预防和惩治腐败工作力度。

各位代表！新时代要有新气象，新时代要有新作为。让我们更加紧密地团结在以习近平同志为核心的党中央周围，高举习近平新时代中国特色社会主义思想伟大旗帜，在市委的正确领导下，同怀愚公志、共尽精卫责，不忘初心、牢记使命、逐梦前行，实干苦干巧干，为加快韶关实现振兴发展、创造更加幸福美好未来而努力奋斗！
